# Vasculitic Rash: A Rare Complication of Klebsiella Liver Abscess in Uncontrolled Diabetes

**DOI:** 10.7759/cureus.97197

**Published:** 2025-11-18

**Authors:** Muhammad Iqbal, Faisal Arslan, Muhammad Kamran, Moaz Ahmar, Syed Muhammad Hadi Moin Jah, Ali Hussain

**Affiliations:** 1 Gastroenterology and Hepatology, Pinderfields Hospital, Wakefield, GBR; 2 Cardiology, Pinderfields Hospital, Wakefield, GBR; 3 Endocrinology and Diabetes, Pinderfields Hospital, Wakefield, GBR; 4 Acute Medicine, Leeds Teaching Hospitals, Leeds, GBR; 5 General Medicine, Hawthorn Drive Surgery, Ipswich, GBR; 6 Acute Medicine, Pinderfields Hospital, Wakefield, GBR

**Keywords:** diabetes mellitus, klebsiella pneumoniae liver abscess, purpuric rash, septic vasculitis, vasculitic rash

## Abstract

*Klebsiella pneumoniae* liver abscess (KPLA) is a known complication in patients with poorly controlled diabetes mellitus, frequently resulting in bacteremia and metastatic infections. However, cutaneous manifestations, such as vasculitic rash, are exceedingly rare. We present a case of a 45-year-old male with uncontrolled diabetes who was admitted with high-grade fever, rigors, and dyspnea. Within 24 h of admission, he developed a bilateral, purpuric, non-blanching rash on the lower limbs suggestive of vasculitis. Laboratory investigations revealed a negative vasculitis screen and positive blood cultures for *Klebsiella pneumoniae* (*K. pneumoniae*). Computed tomography of the abdomen identified a large subcapsular liver abscess. The patient was successfully treated with percutaneous radiologically guided drainage and broad-spectrum antibiotics. This case highlights that clinicians should consider infectious etiologies, such as *K. pneumoniae* in vasculitic rashes among diabetic patients, as misdiagnosis and inappropriate immunosuppression can worsen outcomes.

## Introduction

Klebsiella-related vasculitis is an immune-mediated condition that primarily affects small vessels and typically occurs secondary to *Klebsiella pneumoniae* infection, which commonly enters the bloodstream from the lungs or urinary tract. *Klebsiella pneumoniae* liver abscess (KPLA) is a serious infectious condition, particularly in individuals with diabetes mellitus, where hyperglycemia impairs immune function and increases susceptibility to invasive infections [1,2]. It is frequently associated with bacteremia and metastatic complications, including endophthalmitis, meningitis, and pulmonary involvement [3,4]. Cutaneous manifestations, such as a vasculitic rash, whether from septic vasculitis or leukocytoclastic vasculitis secondary to bacteremia, are exceptionally rare in KPLA [5,6]. This report explores the microbiological features of *K. pneumoniae*, including hypervirulent strains, and outlines the pathogenesis of liver abscess and vasculitic rash in the context of uncontrolled diabetes [7,8].

## Case presentation

A 45-year-old male with poorly controlled type 2 diabetes mellitus (glycosylated hemoglobin {HbA1c} 130 mmol/mol) presented to the emergency department (ED) with a three-day history of high-grade fever, rigors, and progressive dyspnea. He denied recent travel, gastrointestinal symptoms (nausea, vomiting, abdominal pain, or diarrhea), and had no known exposure to infectious contacts. There was no history of sexual activity, and he reported no neurological symptoms, such as headache, neck pain or stiffness, visual disturbances, or altered sensorium. Additionally, he denied joint pain, skin rash, urethral discharge, red eyes, or oral or genital ulcers. His appetite was normal, and there was no history of weight loss.

His past medical history was notable for type 2 diabetes mellitus diagnosed five years earlier, managed with diet and lifestyle modifications. He had not followed up with his primary care provider for routine monitoring or investigations since diagnosis. He reported no known drug allergies and was not taking any regular medications.

On examination, he was febrile (temperature 39.5°C), tachypneic (respiratory rate 28 breaths/min), and tachycardic (heart rate 110 beats/min), with an oxygen saturation of 92% on room air. Blood pressure was 110/70 mmHg. Cardiovascular and abdominal examinations were unremarkable. There were no clinical stigmata of infective endocarditis. Neurological assessment revealed no signs of meningism, and both dermatologic and musculoskeletal examinations were within normal limits.

The patient was clinically septic. Blood cultures were obtained, and empirical antibiotics (intravenous piperacillin-tazobactam three times per day) were initiated for sepsis of unknown origin. Initial laboratory investigations, as shown in Table 1, revealed leukocytosis (white blood cell count: 18.0×10⁹/L; reference range: 4.0-11.0×10⁹/L) and elevated C-reactive protein (CRP: 250 mg/L; reference range: 0-9 mg/L). Liver and renal function tests were within normal limits. Chest radiography was unremarkable.

**Table 1:** Baseline laboratory parameters.

Laboratory parameters	Results	Normal range
Hemoglobin	13.1 g/L	13.0-18.0 g/L
White blood cells	18×10⁹/L	4.0-11.0×10⁹/L
Neutrophils	21.1×10⁹/L	1.5-8.0×10⁹/L
Platelets	298×10⁹/L	150-450×10⁹/L
CRP	250 mg/L	0-9 mg/L
Alanine aminotransferase (ALT)	16 U/L	0-56 U/L
Alkaline phosphatase (ALP)	102 U/L	30-130 U/L
Bilirubin	5 μmol/L	0-21 μmol/L
Albumin	31 g/L	32-52 g/L
HbA1c	14%	<5.6%
Blood glucose	225 mg/dL	70-135 mg/dL
Urea	17.1 mg/dL	6-24 mg/dL
Creatinine	1.1 mg/dL	0.7-1.2 mg/dL

**Figure 1:**
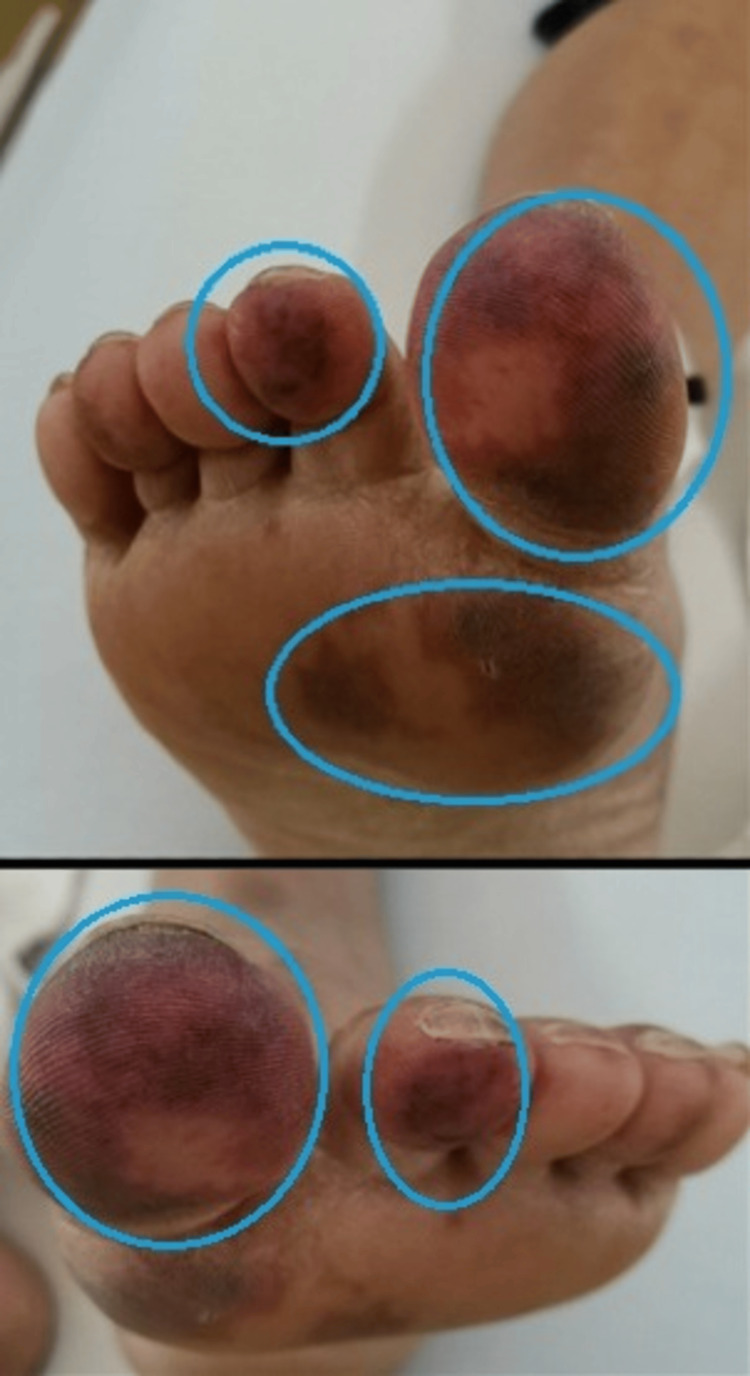
Rounded areas show a bilateral, purpuric rash on both feet.

**Figure 2:**
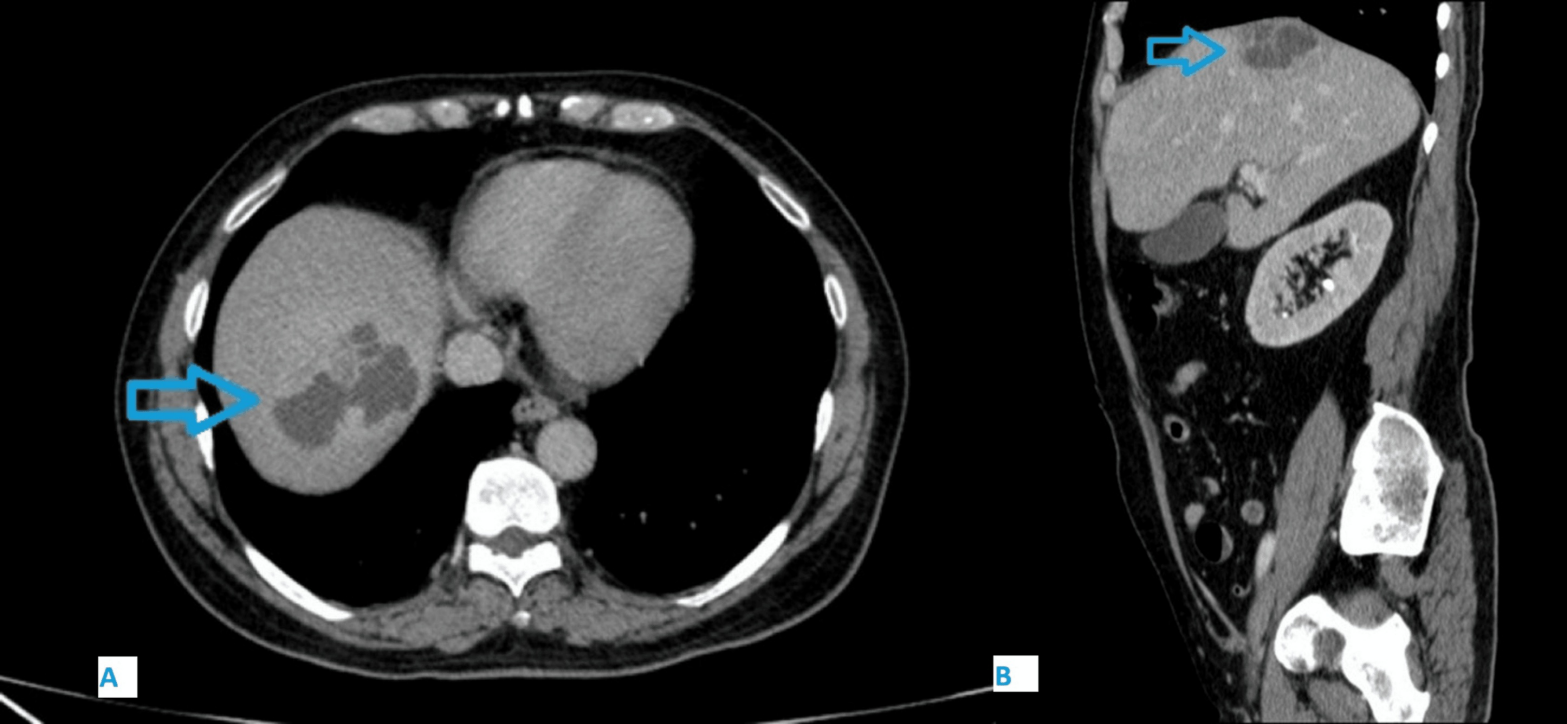
Abdominal CT images showing a liver lesion. (A) Axial view and (B) sagittal view CT of the abdomen reveals an irregular fluid-density lesion within the liver, demonstrating rim enhancement and a thin outer ring of hypoattenuation, suggestive of a liver abscess (indicated by arrows).

Twenty-four hours post-admission, the patient developed a purpuric, non-blanching rash on both feet, extending from the big toe to the ankle joints bilaterally (10x4 mm in size), suggestive of small-vessel vasculitis. The rash was palpable, symmetrically distributed, and spared mucous membranes and other sites (Figures 1, 2A, 2B).

After a new rash, to exclude ischemia, examination of the peripheral pulses of the lower limbs was performed, which showed good volume and perfusion with normal overlying skin temperature and preserved sensation. A bedside Doppler was performed, which was unremarkable. Furthermore, there were no clinical stigmata of infective endocarditis. The transthoracic echocardiogram was negative for any evidence of infective endocarditis. A thorough examination for tick or insect bites was unremarkable. A comprehensive vasculitis screen, as shown in Table 2, including antinuclear antibody (ANA), anti-neutrophil cytoplasmic antibody (ANCA), and rheumatoid factor, returned negative.

**Table 2:** Vasculitis screen panel. ANA: antinuclear antibody; ANCA: anti-neutrophil cytoplasmic antibody; LKM: liver-kidney microsomal

Antibody	Results
ANA	Negative
Cytoplasmic ANCA	Negative
Perinuclear ANCA	Negative
Rheumatoid factor	Negative
Anti-smooth muscle antibody	Negative
Anti-LKM antibody	Negative
Anti-gastric parietal cell antibody	Negative
Anti-mitochondrial antibody	Negative
Anti-ribosomal antibody	Negative

Urgent lower limb Doppler ultrasound excluded vascular insufficiency and deep vein thrombosis. A computed tomograph (CT) of the chest, abdomen, and pelvis was done, which showed a 6x7x6 cm subcapsular liver abscess in segment 8 of the liver (Figures 2A, 2B). Despite 48 h of intravenous antibiotics, the patient showed no clinical improvement. The microbiology team recommended radiologically guided drainage. Percutaneous drainage under ultrasound guidance yielded purulent material, with cultures confirming *K. pneumoniae* with identical susceptibility patterns. Treatment continued with broad-spectrum antibiotics, including metronidazole (500 mg every 8 h) for anaerobic coverage and vancomycin (1 g every 12 h), alongside insulin therapy for glycemic control.

**Figure 3:**
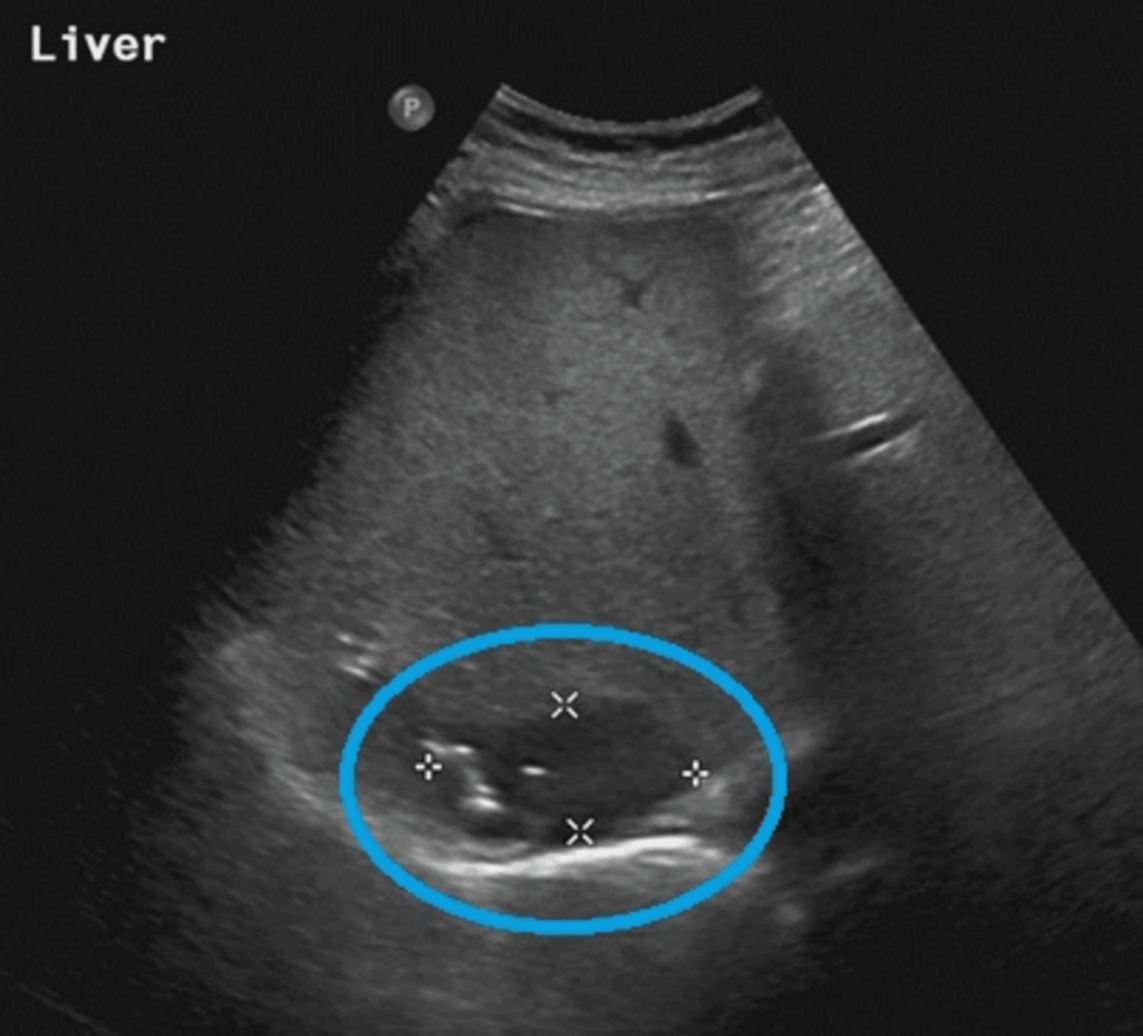
Ultrasound liver shows marked reduction of size of abscess (indicated by circle).

Within 48 h post-drainage, the patient’s fever resolved, and the vasculitic rash gradually subsided over one week without immunosuppressive therapy. A follow-up liver ultrasound two weeks later showed a significant reduction in the abscess, 3x2 cm (Figure 3). The patient was discharged after seven days of oral antibiotics and optimized diabetic management, with no recurrence at three-month follow-up.

## Discussion

*Klebsiella pneumoniae*, a Gram-negative, encapsulated, non-motile, rod-shaped bacterium of the Enterobacteriaceae family, is a commensal in the human gastrointestinal and oropharyngeal tracts [7]. While typically an opportunistic pathogen causing nosocomial infections like pneumonia and urinary tract infections, hypervirulent strains (capsular serotypes K1 and K2) are implicated in community-acquired invasive syndromes, including pyogenic liver abscess. Hypervirulent strains are characterized by a hypermucoviscous phenotype and specific virulence factors such as the rmpA gene and siderophore biosynthesis genes, enabling invasive infections in both immunocompromised and healthy hosts [8].

In diabetic patients, KPLA pathogenesis involves host susceptibility and bacterial virulence. Hyperglycemia impairs neutrophil chemotaxis, phagocytosis, and oxidative burst, facilitating bacterial overgrowth in the gut [1,7]. Klebsiella translocates across the intestinal barrier, possibly via impaired tight junctions or microaspirated oropharyngeal colonization, entering the portal venous system and seeding the liver [7,8]. The bacterium’s antiphagocytic capsule enables evasion of hepatic Kupffer cell clearance, leading to suppurative necrosis and abscess formation. Bacteremia may promote metastatic spread [8].

Cutaneous vasculitic manifestations, such as the purpuric rash in this case, likely result from septic embolization or immune-mediated mechanisms during *K. pneumoniae* bacteremia [5]. Septic vasculitis typically involves non-immunologically mediated vessel damage with thrombosis and minimal leukocytoclasis due to direct microbial effects, whereas immune complex-mediated leukocytoclastic vasculitis features prominent leukocytoclasis, fibrinoid necrosis, and immune deposits triggered by complement activation and neutrophil recruitment [9]. Leukocytoclastic vasculitis (LCV) arises from immune complex deposition containing bacterial antigens in post-capillary venules, which activates complement and recruits neutrophils. This causes damage to the vessel wall, fibrinoid necrosis, and red blood cell extravasation, manifesting as purpura [5,6]. Hypervirulent *K. pneumoniae* strains may exacerbate this by inducing high-level bacteremia and immunostimulatory lipopolysaccharides, thereby promoting cytokine storms and endothelial dysfunction [6]. LCV typically resolves with source control and antibiotics, as seen here, without requiring immunosuppression [5].

KPLA is increasingly prevalent in diabetic populations due to impaired immunity in hyperglycemic states [1,2]. Poor glycemic control (HbA1c of 130 mmol/mol), as in this case, heightens the risk of invasive syndromes, including bacteremia and metastases [3,4]. While endophthalmitis and pulmonary abscesses are common complications, cutaneous vasculitis is rare and often linked to septic emboli or immune complex deposition [5,6]. The temporal association between bacteremia, rash onset, and negative autoimmune markers supports an infectious etiology. Rash resolution following source control and antibiotics further confirms this. Management of KPLA requires prompt drainage and targeted antimicrobials, with initial broad-spectrum coverage for potential polymicrobial involvement [3,4]. Optimizing glycemic control is critical to prevent recurrence, as reported in the literature [1].

Limitations of this study include the lack of a skin biopsy to confirm LCV histologically, despite characteristic clinical features. Although a skin biopsy is ideal, it is often unnecessary when the rash resolves quickly after treatment for the infection. Future studies should explore the interplay of hyperglycemia, *K. pneumoniae* virulence, and vasculitis complications.

## Conclusions

This case highlights vasculitic rash as a rare complication of *Klebsiella pneumoniae* liver abscess in uncontrolled diabetes, driven by hypervirulent strains and impaired immunity. Pathogenesis involves bacterial translocation, abscess formation, and bacteremia, leading to immune complex-mediated vasculitis. Early recognition of atypical presentations, such as a purpuric rash, is crucial for timely intervention.

Clinicians should consider infectious etiologies for vasculitic rashes in febrile diabetic patients, prioritizing antimicrobial therapy over immunosuppression. Optimizing glycemic control is essential to reduce the risk of recurrence and morbidity. Future research on *K. pneumoniae* virulence factors and host-pathogen interactions may enhance prevention strategies.
